# Conductive Plastics from Al Platelets in a PBT-PET Polyester Blend Having Co-Continuous Morphology

**DOI:** 10.3390/polym14061092

**Published:** 2022-03-09

**Authors:** Abdullah Alhamidi, Arfat Anis, Saeed M. Al-Zahrani, Zahir Bashir, Maher M. Alrashed

**Affiliations:** SABIC Polymer Research Center (SPRC), Chemical Engineering Department, King Saud University, P.O. Box 800, Riyadh 11421, Saudi Arabia; akfhk90@hotmail.com (A.A.); szahrani@ksu.edu.sa (S.M.A.-Z.); zbashir2703@gmail.com (Z.B.); mabdulaziz@ksu.edu.sa (M.M.A.)

**Keywords:** reinforced polymer composite, metal–plastics, PET/PBT blend, mechanical properties, conductive plastics

## Abstract

Conductive plastics are made by placing conductive fillers in polymer matrices. It is known that a conductive filler in a binary polymer blend with a co-continuous morphology is more effective than in a single polymer, because it aids the formation of a ‘segregated conductive network’. We embedded a relatively low-cost conductive filler, aluminium nano platelets, in a 60/40 PBT/PET polymer blend. While 25 vol.% of the Al nanoplatelets when placed in a single polymer (PET) gave a material with the resistivity of an insulator (10^14^ Ωcm), the same Al nano platelets in the 60/40 PBT/PET blend reduced the resistivity to 7.2 × 10^7^ Ωcm, which is in the category of an electrostatic charge dissipation material. While PET tends to give amorphous articles, the 60/40 PBT/PET blends crystallised in the time scale of the injection moulding and hence the conductive articles had dimensional stability above the T_g_ of PET.

## 1. Introduction

There is a demand for thermally and electrically conductive plastics due to applications in electronics, and the emerging electric car segment. Housings for electronics can be built of metal, but where mobility is involved in the application [1], light-weighting is also desired, and then conductive plastics would be the solution. Ordinary plastics with an electrical conductivity of ~10^−11^ to 10^−21^ S/m (electrical resistivity of ~10^11^ to 10^21^ Ωm) cannot be used for housing electronics due to electromagnetic interference (EMI) and radio frequency interference from nearby devices. For EMI shielding, conductive plastics with a resistivity in the range of 1 Ωm or lower are needed. Conductive plastics with a resistivity of 10^2^ Ωm to 10^7^ Ωm are enough for electrostatic charge dissipation (ESD). Thermally conductive composites are also in demand for heat dissipation and heat management in electronic packaging, in light-emitting diode (LED) assemblies, and for the emerging electrical car sector. Whereas metals may have thermal conductivity in the range of ~200–400 W/m·K, commercial conductive composites reach only about 1–10 W/m·K, but this is sufficient for many applications.

Conductive plastics must meet the following criteria: (1) they must reach adequate electrical or thermal conductivity as needed by the application, (2) have acceptable mechanical properties, particularly the strength and impact, (3) be easy to process, and (4) have an acceptable cost. Fire retardancy is compulsory for some electrical applications. The search for conductive plastics continues as it is difficult to meet all the criteria simultaneously. Carbon black is the earliest and still most widely used conductive filler [2,3,4].

Some of the principles behind conductive plastics have been established [5,6,7,8]. The filler shape (spherical, platelet, fibre, or irregular particles) plays a role in the critical volume fraction for percolation, with fibre or rod-like particles requiring the lowest amount. Commercial conductive plastics for EMI shielding use steel micro fibres [6]. Random or homogeneous distribution of the filler is undesirable as it requires high filler loadings such as 30–40 volume (vol.%) for establishing contact for a conductive network. Mamunya et al. [7] showed that when polyvinyl chloride (PVC) powder and finer metal powder were mixed and compression moulded, a composite with a ‘segregated network’ (also called a heterogeneous network) was formed; the metal particles were collected between the boundaries of polymer particles, and this allowed conductivity at lower volume fractions. For extruded conductive composites, Sumita et al. [9] showed that the ‘segregated network’ can be obtained by using carbon black conductive filler in an immiscible binary polymer blend (instead of in single polymers). Immiscible blends with about equal amounts of two polymers form a co-continuous morphology and the interfacial positioning of the conductive filler is ideal for segregated network formation because the interface area is small, and it leads to the possibility of ‘double percolation’ [9]. This concept of a ‘segregated network’ formed by a conductive filler in polymer blends with co-continuous morphology has been demonstrated, for example, by Li et al. [10] with carbon black in a co-continuous PBT/polyamide 6 blend and Bai et al. [11] with graphene in a co-continuous blend of polylactide and poly(ethylene-*co*-vinyl acetate).

Another concept that is currently being explored to achieve electrical and/or thermal conductivity with lower filler content is to use ‘hybrid fillers’, for example, graphite particles + carbon fibres (Thongruang et al. [12]), carbon black + short carbon fibres (Leng et al. [13]), and CNTs + graphene (Perets et al. [14]). Two synergistic conductive nets are formed by carbon black particles and carbon fibres [13], because the fibrous filler interacts with spherical particles of carbon black, and a similar principle would operate with a hybrid filler system of CNTs and graphene [14].

Gao et al. [15] combined both concepts, where a hybrid filler (up to 10 parts per hundred (phr) of graphene + CNTs) was placed in a polymer blend of 70/30 PBT/PET. However, there was a reduction in mechanical properties (the notched Izod impact was about 30–50% lower) compared to the unfilled blend.

CNTs and graphenes are expensive and the surface treatments proposed are often impractical [16]. At current prices, multi-walled CNT powder is ~50x and graphene is ~100x the price of aluminium powder. Hence, in a recent paper, we revisited metal fillers for conductive plastics [17]. We found that aluminium-poly(ethylene terephthalate) (PET) had a naturally strong adhesion and gave good mechanical properties [18]. However, in the works with Al-PET using Al nodular particles [17] and Al nano platelets [18], while mechanical properties were not impaired, even at the highest extrudable volume percentage (25 vol.% of Al) the percolation threshold was not breached; the filled plastic remained in the insulator regime of electrical resistivity.

In light of the research showing that polymer blends rather than single polymers are better for creating the segregated network [9], in this work, we decided to incorporate the Al platelets in a poly(butylene terephthalate) (PBT)-PET polyester blend with a composition (60/40 by weight) which should give a co-continuous immiscible blend, to see if this would allow percolation for electrical conductivity. There was a second reason for using PBT-PET instead of PET. PET is a slowly crystallising polyester and this means injection moulded articles will be amorphous if cold moulds are used [17,18]. PBT is able to crystallise up to a cooling rate of ~300 K/s, while PET can crystallise only if the cooling rate is slow and is limited to ~2 K/s or lower; the 60/40 PBT/PET blend can crystallise up to an intermediate cooling rate of around 50 K/s [19]. If the moulded article is amorphous, it will soften and lose shape above the T_g_, whereas a semi-crystalline article is limited by its T_m_.

Here, we demonstrate that the same Al platelets which showed electrical insulator behaviour with 25 vol.% in amorphous PET [18] led to a conductive plastic in the electrostatic charge dissipation range, in a 60/40 PBT/PET co-continuous blend. The mechanical properties were acceptable and since the as-moulded articles from the blend were semi-crystalline instead of amorphous, they showed resistance to shrinkage and warping above the T_g_ of the PET.

## 2. Experimental

### 2.1. Materials

The matrix was chosen to be 60/40 PBT/PET. Polybutylene terephthalate (PBT) was supplied by Sipchem Chemical Company, Al Khobar, Saudi Arabia (Grade: PBT-R1-G0-011). It is a high-viscosity PBT resin and it has a density of 1.31 g/cm^3^. Polyethylene terephthalate (PET) granules were supplied by the Saudi Basic Industries Corporation (Al-Jubail, Saudi Arabia) (SABIC) (Bottle Grade PET BC 212). It was semi-crystalline, with an intrinsic viscosity of 0.84 dL/g. Aluminium powder in nano platelet form (Nanografi Nano Technology, Thuringen, Germany) was used as the conductive filler. The platelets or flakes were 5–10 μm in lateral width and 50–100 nm in thickness [17]. The platelets did not have any polygonal shape; they were irregular [18].

### 2.2. Preparation of Al-PBT/PET Composites

**Figure 1:**
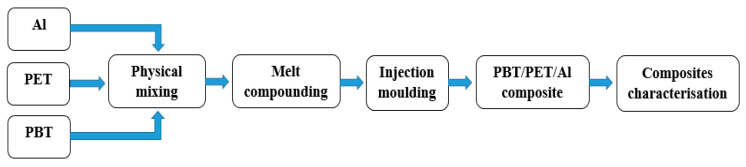
Schematic diagram of the composite preparation process.

Figure 1 shows the schematic of the composite preparation process. Table 1 shows the compositions of Al and PBT/PET.

The weight ratio of the PBT/PET matrix was fixed at 60/40. The pellets of PET and PBT were dried in an oven at 120 °C for 24 h prior to the melt compounding to reduce hydrolysis during the melt mixing process. PET and PBT pellets (in the ratio 60:40 PET:PBT) and Al nano platelet powder were mixed together physically. They were fed to a DSM Xplore micro compounder, 15 cc (Sittard, The Netherlands) with co-rotating twin screws. The operational conditions were 3 mins of mixing time with a temperature of 260 °C at a screw speed of 100 rpm. The extruded composite melt from the mini twin screw extruder was collected in a pre-heated collector equipped with a cylindrical piston assembly. The collector temperature was kept the same as the micro compounder temperature (260 °C). The collected melt was then introduced to a moulding machine (DSM Xplore micro injection moulder, IM 12 cc, Sittard, The Netherlands), for the preparation of dog-bone-shaped bars for a tensile test, rectangular bars for Izod impact and flexural tests, and square-shaped plaques for the thermal and electrical conductivity measurements.

The mould temperature and holding pressure were kept at 24–26 °C and 6 bar, respectively, with a total injection time of 45 s.

### 2.3. Characterisation of Composites

#### 2.3.1. Scanning Electron Microscope (SEM)

The morphology of the Al-60/40 PBT/PET bar was characterised with a field emission scanning electron microscope (FE-SEM), (Model: JEOL JSM-7600F, JEOL Ltd., Tokyo, Japan) at an accelerating voltage of 5 kV. The cryo-fracture surface of the bars was imaged. This gave information on the orientation and spatial distribution of the platelets in the composites. The fractured bars were mounted on 0.5-inch pin stubs using a carbon adhesive tape and then coated with a thin layer of gold for 30 s, to prevent any charging.

#### 2.3.2. X-ray Diffraction (XRD)

We desired the matrix of the moulded bars of the Al composite, in this case the 60/40 PBT/PBT, to be semi-crystalline rather than amorphous, so that articles made from them would be capable of crossing the glass transition temperatures of the two polymers without distortion. To check the long-range order in the matrix, wide angle X-ray diffraction (XRD) patterns of the unfilled PET, PBT, 60/40 PBT/PET bars and the Al-filled 60/40 PBT/PET bars were recorded with Cu-Kα radiation (Bruker D8 Discover, Karlsruhe, Germany). The results were collected over a 2θ range of 5–85°, with continuous scan mode at 20 kV voltage and 5 mA current. The dwell time and the step size of the scanned range were maintained at 3 s and 0.2°, respectively, with scanning rate of 4°·min^−1^.

#### 2.3.3. Differential Scanning Calorimetry (DSC)

Melting and crystallisation behaviours of the PET, PBT, the 60/40 PBT/PET blend, and the Al/PBT/PET bars were examined by DSC. A DSC-60A (Shimadzu, Tokyo, Japan) thermal analyser was used. The samples were heated from 30 to 280 °C with a heating rate of 10 °C/min, then kept at a temperature of 280 °C for 3 min, and then cooled to 30 °C at 10 °C/min. The analysis of the T_g_ and the presence or absence of a cold crystallisation exotherm in combination with the X-ray pattern allowed us to resolve fine issues related to the order (skin–core morphology) in the bars.

#### 2.3.4. Shrinkage Measurements after Thermal Exposure above the T_g_ of PET

The T_g_ of PET is 78 °C. The shrinkage in length of the as-moulded bars of amorphous PET, 20 vol.% Al-PET, 60/40 PBT/PET, and 20 vol.% Al-60/40 PBT/PET was measured after annealing at 150 °C in an oven for 30 min. Flexural bars were cut to a uniform length of 60 mm and then the % shrinkage was calculated. The experiments were performed two times and the length after heat exposure was measured.

#### 2.3.5. Tensile Test

The tensile test was carried out using a Tinius Olsen uniaxial universal testing machine (Model: H100KS, Horsham, PA, USA). The tensile bars had the following dimensions: end-to-end length L = 150 mm, width W = 12.7 mm × depth D (thickness) = 3.25 mm. Our tensile bar’s dimensions were similar but not identical to the Type I bar of ASTM D638-14 (Standard Test Method for Tensile Properties of Plastics). The only difference, however, was that our end-to-end length was shorter (in the ASTM Type I, the end-to-end length L = 165 mm instead of 150 mm). That is, in our bar, the grip section was shorter than in the ASTM bar by 7.5 mm. This was because the shot size of the mini injection moulder could not accommodate the volume needed to make two ASTM Type 1 bars in a single shot, with the full end-to-end length of 165 mm. However, the use of a shorter grip has no influence on the measurement, and as all other features including the gauge length and the radius of curvature of the filet were the same, we say our tensile bar was in essence similar to the Type I tensile bar of ASTM D638-14. The following critical portions in our bar were identical to the ASTM D638-14 Type I bar: the length of the narrow section of the bar was 57 mm and the gauge length was 55 mm; the radius of curvature of the curved portion (filet) which joins the narrow part of the bar to the grip portion was 76 mm. A test speed of 50 mm/min was used. ASTM D638-14 recommends using 5 mm/min except when the test takes more than 5 min, in which case 50 or 500 mm/min is allowed. With PET, PBT, and PBT-PET, the extension-to-break was very high, and the test takes more than 5 min, hence, we selected 50 mm/min. Tensile modulus, tensile strength, and strain at break (elongation) were determined. The reported values are an average of 7 measurements. Further details of the tensile test are shown in the Supplementary Information.

#### 2.3.6. Flexural Test

The three-point flatwise bending flexural test was performed using a Tinius Olsen uniaxial universal testing machine (Model: H100KS, Horsham, PA, USA). The support span length was 52 mm and a crosshead speed of 5.2 mm/min was used, conforming to the ASTM D 790-03 standard with procedure B. The bar’s length was 134 mm, the width was 12.7 mm, and the depth (thickness) was 3.25 mm. The Supplementary Information gives more details of conformance to ASTM D 790-03 (bar dimensions, span, overhang, cross head speed, conditioning, etc.). Flexural properties such as flexural modulus, flexural strength, and strain at maximum stress were determined. The reported values of flexural modulus and flexural strength are an average of measurements performed on 7 samples.

#### 2.3.7. Notched Izod Impact Test

The notched Izod impact test was used to evaluate the impact resistance of the Al-PBT/PET composites by using an AMSE pendulum impact tester machine (Torino, Italy), according to the ASTM D 256-04, Type A test. The values for amorphous PET, PBT, and unfilled 60/40 PBT/PET were also measured. The moulded bar’s dimensions were length of 64 mm, width of 12.7 mm, and thickness of 3.25 mm as set by the standard. It was notched by machine. The specimens were notched in the middle of the sample at a distance of 31.8 mm (half way along the length), the notch depth was 2.5 mm with a radius of curvature of 0.25 mm, and the remaining depth was 10.16 mm ± 0.05. The notched bar was then placed vertically in the vice with the notch facing toward the pendulum and it was hit using a swinging hammer with an energy of 5.5 J and impact velocity of 3.50 m/s. At least ten specimens were tested for each composite to ensure repeatability. The amorphous PET, the PBT, 60/40 PBT/PET, and all the Al-60/40 PBT/PET bars broke according to the Type C break (complete break) mentioned in the standard—that is, on break, the bar separated into two pieces. Hence, as per the standard, the impact value comparisons are valid as the failure category was the same for all compositions. The Supplementary Information gives more details of conformance to ASTM D 256-04.

#### 2.3.8. Electrical Resistivity Measurement

The resistivity measurements were taken on injection-moulded plaques according to ASTM D257, using a Keithley electrometer/high-resistance meter (Model 6517B) coupled with a resistivity test fixture (Model 8009) wherein the electrodes are located in a shielded box to minimise stray electrostatic pick-up, which can cause measurement errors. The electrodes were made of stainless steel and were built to ASTM standards. The electrodes in the test fixture were coated with conductive rubber for better sample–electrode contact. The measurements were performed using the alternating polarity method which is designed to improve high resistivity measurements. Resistivity measurements are prone to large errors due to background currents. By using an alternating stimulus voltage, it is possible to eliminate the effects of these background currents. The voltage applied for the resistivity measurements was 100 V. All measurements were done in triplicate.

#### 2.3.9. Thermal Conductivity Characterisation

The thermal conductivity values of the 60/40 PBT/PET blend and its composites were measured in triplicate using a TCi thermal conductivity analyser from C-Therm Technologies (Fredericton, NB, Canada). It uses a modified transient plane source sensor conforming to ASTM D7984 for the thermal conductivity measurements. The reported values in W/m·K are an average three different measurements through the thickness at ambient temperature. The test specimens were square plaques that were injection moulded.

## 3. Results and Discussion

### 3.1. The Nano Aluminium Platelets

Li and Chung [20] investigated fibres of carbon, nickel, and steel and Al flakes in polyethersulphone (PES), and found the Al flakes gave the best combination of electrical conductivity and mechanical properties. The latter was ascribed to the good adhesion of Al flakes with PES.

Al powders are commonly available with the following particle shapes: (1) nodular or irregular, (2) spherical, (3) flakes/platelets. Fibre form is also available but chopped Al fibres could not be sourced. Nodular and spherical shapes are made by gas atomisation and, depending on the gas, the particles may be spherical or nodular. Both micron size and nano size are available. Flakes are made by ball milling of the spherical powder to flatten them into platelets.

**Figure 2:**
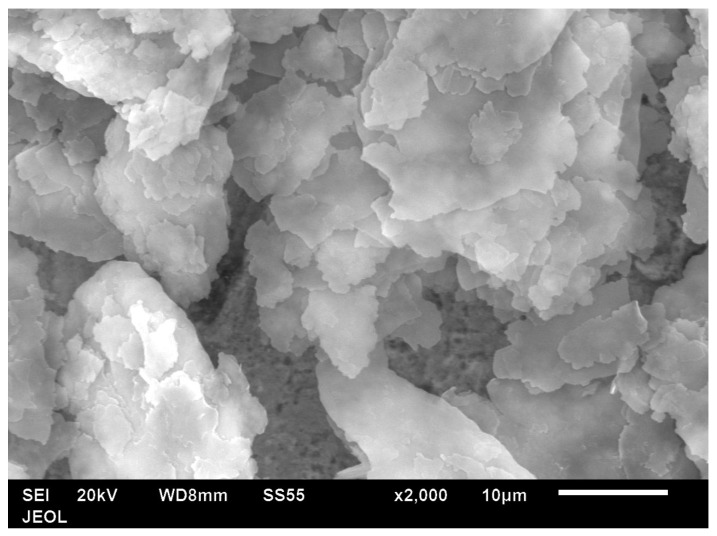
SEM image of Al nano platelet particles.

The manufacturer (Nanografi) did not show any characterisation data apart from an indication that the Al was flakes. Figure 2 shows that these flakes were 5–10 microns in width. The average thickness was established as ~65 nm in previous work where the same platelets were incorporated into PET [18]. As one dimension is nano, we can regard these as nano platelets of Al. The platelets are not regular in the sense that they are not a particular shape such as hexagonal or square (Figure 2). The Al flakes used by Li and Chung [20] in polyethersulphone (PES) had facial dimensions of ~1 mm and the particles were quite rectangular in shape. Further, they noted that one face of their Al flake had been roughened, while the opposing face was smooth. With such a large platelet size, the Al-PES composites would have been unextrudable, so they were compression moulded [20].

### 3.2. Skin–Core Morphology in Injection-Moulded PBT and 60/40 PBT/PET Bars

**Figure 3:**
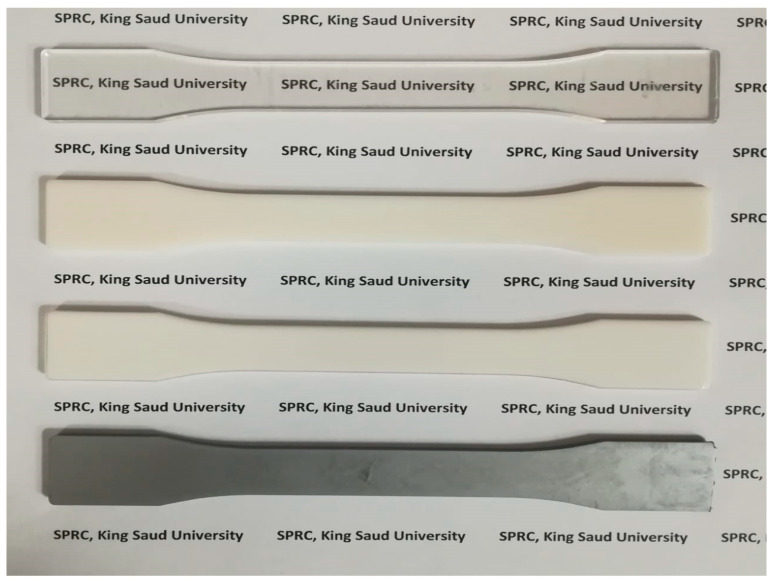
Injection-moulded tensile bar appearance of polymers and composites. From top to bottom: amorphous PET bar is transparent; semi-crystalline PBT bar is white; 60/40 PBT/PET bar is cream tinted; and the Al nano platelet-filled 60/40 PBT/PET bar is silvery.

Figure 3 shows the appearance of the injection-moulded bars made with cold moulds. The PET bar (top) was uniformly amorphous and transparent throughout the thickness. The PBT bar in contrast was white due to spherulitic crystallisation (Figure 3, second from top). It had a yellow tint due to the titanium compound used as a catalyst. The 60/40 PBT/PET bar was also off-white but was more translucent than the PBT (Figure 3, third from top).

**Figure 4:**
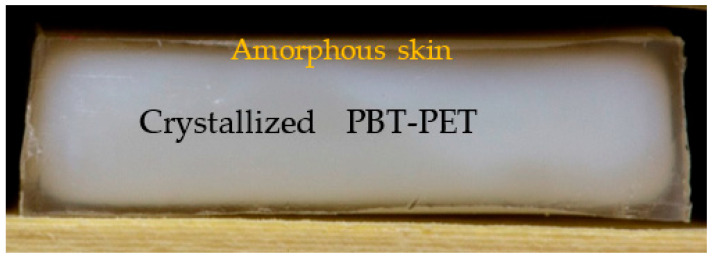
Cross-section of the 60/40 PBT/PET bar shows a transparent jacket. The horizontal width of the bar is 12.7 mm.

Closer inspection by eye of the 60/40 PBT/PET bar showed a transparent skin, about 500 mm thick, which jacketed all faces of the bar. This is not easily visible in Figure 3. The cross-section of a cut bar in Figure 4 clearly shows the transparent jacket. The core part of the 60/40 PBT/PET bar was opaque. A similar effect was found in the PBT bar except the transparent skin was less apparent as it was thinner (100 μm).

Hobbs and Pratt [21] showed that even in the faster crystallising PBT, there can be a skin–core, with the non-spherulitic, amorphous skin ranging in thickness from 20–200 μm depending on the melt injection temperature and mould temperature (21 °C and 121 °C). Such skins can also occur with other fast-crystallising polymers. Spoerer et al. showed a 40 μm deep amorphous skin in injection-moulded polyamide 66 [22]. Hnatkova and Dvorak showed a 124 μm to 416 μm amorphous skin in injection-moulded polypropylene (PP) [23].

Polarised light microscopy (not shown) indicated that the transparent skin of the 60/40 PBT/PET bar was non-birefringent and was about 425–500 μm thick. It was made of amorphous 60/40 PBT/PET.

### 3.3. The Skin–Core Also Exists in the 60/40 PBT/PET with Al Platelets

**Figure 5:**
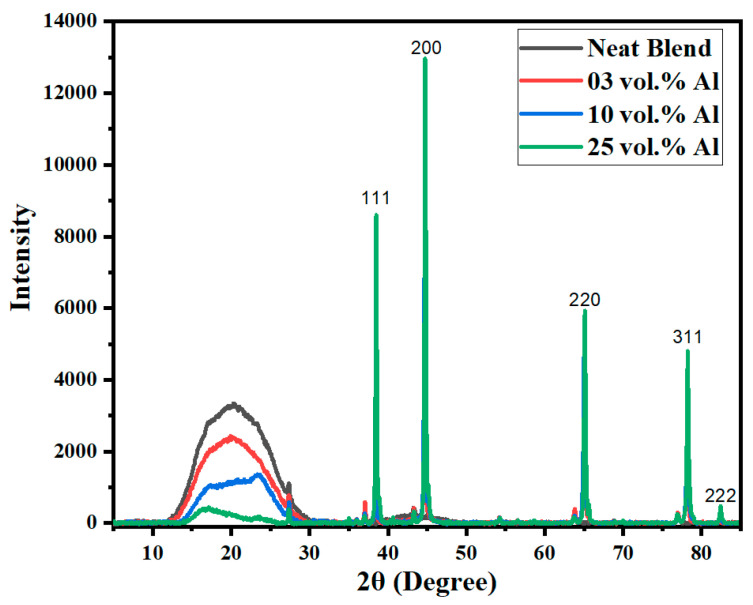
Wide-angle X-ray diffractograms for bars of Al nano platelet 60/40 PBT/PET composites showing the polymer and Al peaks. The polymer blend shows an amorphous halo in the skin portion of the bar. The intense peaks with the Miller indices are from the aluminium. Note, however, that the small sharp peaks at 2θ ~27°, 37°, 44°, 64°, and 77° are artefacts from the sample holder.

Now, we consider the 60/40 PBT/PET blends with the Al nano platelets. The bottom bar in Figure 3 shows the PBT-PET blend with Al platelets. The bar was silvery. One may expect that the 60/40 PBT/PET bar with Al would also have an amorphous skin like the 60/40 PBT/PET bar, which cannot be seen by eye in the silvery bar, due to the aluminium. In the Al-60/40 PBT/PET bars, there are two opposing factors that would determine the depth of the amorphous skin. The Al particles increase the thermal conductivity of the melt and hence would aid the quenching of the material adjacent to the metallic mould wall, and this would increase the skin’s depth. On the other hand, our previous works [18] showed that the Al acts as a nucleating agent for the crystallisation of PET melt, and this could reduce the amorphous skin’s depth. As thermal conductivity and hence thermal diffusivity depend on Al content (shown later), the skin’s depth could depend on the Al content. From the X-ray diffractograms of the as-moulded bars (Figure 5), we can deduce the existence of an amorphous skin of substantial thickness in the 60/40 PBT/PET blends with Al platelets. Figure 5 shows in the region of 2θ extending from 11.5 to 31.0°, where the crystalline peaks of PET and PBT should be observed, there is a broad amorphous halo. The same was observed in the as-moulded 60/40 PBT/PET bar. When the 60/40 PBT/PET bar was annealed at 170 °C, the skin crystallised, and the 2θ range extending from 15–30 °C showed overlapping peaks from semi-crystalline PET and PBT. Alternatively, if the 425–500 μm skin of the as-moulded 60/40 PBT/PET bar was shaved off, the X-ray diffractogram showed overlapping peaks from semi-crystalline PET and PBT, indicating the two polymers in the core of the bar had crystallised (see Supplementary Information, Figure S1).

The reason why the as-moulded bars without and with Al (Figure 5) showed an apparent lack of crystallinity in the polymer matrix is due to the amorphous skin. The intensity attenuation depth or penetration depth of the X-ray in plastics is only a few hundred microns. The skin of the 60/40 PBT/PET bar was 500 μm thick and hence only the long-range order in the skin was seen, and this was amorphous. The fact that the as-moulded Al-60/40 PBT/PET bars also showed a diffuse halo at 2θ extending from 11.5 to 31.0° (Figure 5) means there was an amorphous skin present that was invisible by eye, but it was difficult to detect its depth by microscopy.

From 2θ = 35–100°, sharp crystalline peaks were found at 2θ values of 38.40°, 44.60°, 65.04°, 78.08°, and 82.28° and these were matched [18] to the peaks from the aluminium powder (Figure 5). Metals have even shallower depth of penetration of the X-ray beam (~20 μm), therefore the appearance of the aluminium peaks from the surface of the unshaved bar confirms the platelets are well distributed in the skin as well, regardless of the amorphous matrix skin’s depth.

**Figure 6:**
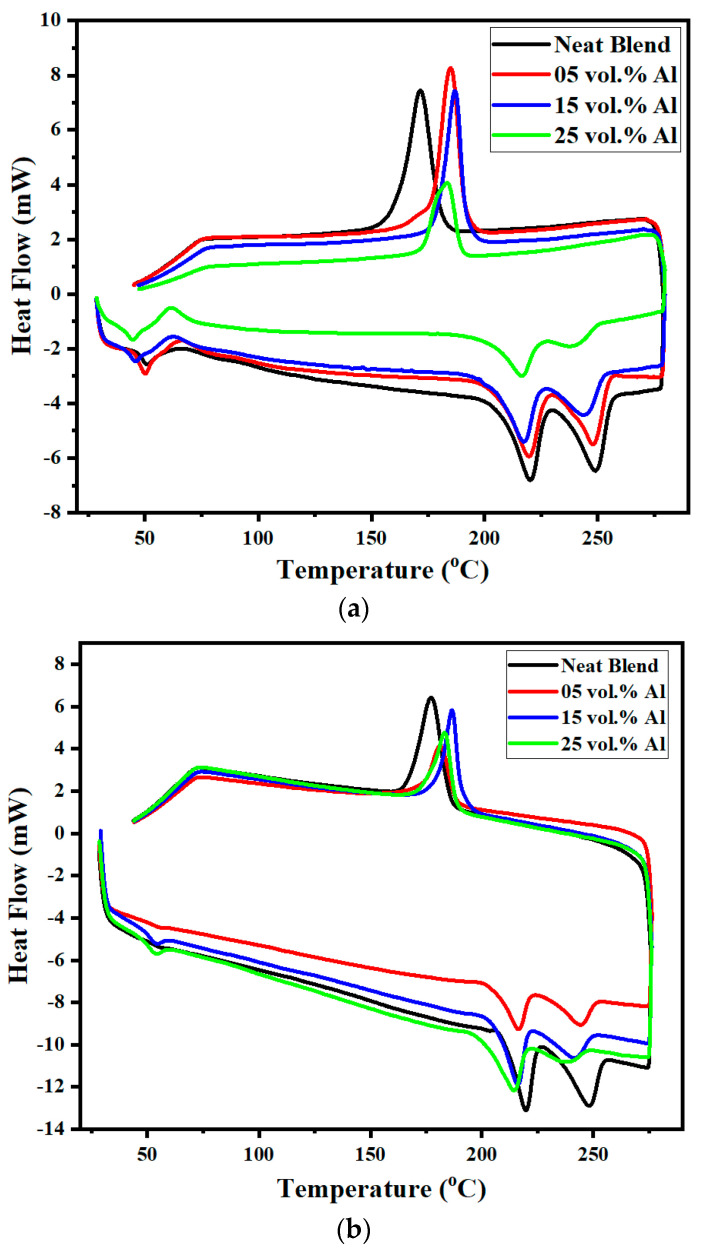
(**a**) DSC of the shaved skins of 60/40 PBT/PET and Al-60/40 PBT/PET bars shows a cold crystallisation exothermic peak at ~60 °C after the T_g_, at ~50 °C, during heating. It indicates there is an amorphous skin in all of them; the arrow indicates the exothermic cold crystallisation during the scan. (**b**) DSC of 60/40 PBT/PET and Al-60/40 PBT/PET (core of moulded bars after shaving off the skin) shows no cold crystallization after the T_g_. This indicates the polymers in the core of the bars had crystallized during moulding.

The DSC in Figure 6a of as-moulded bars of the 60/40 PBT/PET composites with Al platelets also indirectly indicates the existence of an amorphous skin (transparent part in Figure 4), but confirms that both the PET and PBT domains in the core of the blend bar were crystallised. The DSC curves in Figure 6a resemble those of the amorphous PBT-PET blends made by liquid nitrogen ultra-quenching by Avramova [24]. It has the following features: there is a T_g_ at ~50 °C (corresponding to that of PBT), followed by a broad cold crystallisation exotherm (arrowed in Figure 6a), which may hide the T_g_ of PET, this is then ensued by the separate melting of PBT and PET. The latter indicates that the two polymers crystallise, but in separate domains. On the other hand, in the cooling curve after melting, there is remarkably and intriguingly only a single crystallisation peak as reported by others, which is taken as indicating simultaneous yet separate crystallisation of the PET and PBT [24]. Even when Al is present, Figure 6 shows remarkably that the crystallisation peak from the melt is still single but it shifts to higher temperatures, indicating that the Al acts as a nucleating agent.

Figure 6b shows the DSC curves of the core part of the as-moulded Al-60/40 PBT/PET bars and the 60/40 PBT/PET bars. In contrast to Figure 6a, the core part (which appears white in Figure 4) of the blend and its Al composites showed an endothermic blip due to the T_g_, but no cold crystallisation exothermic peak after the T_g_, because the white inner part of the bar had already crystallised during moulding. The arrowed exothermic peaks of Figure 6a seen in the amorphous skin are not seen in the crystallised core of the bar.

The crystallinities of the PET and PBT domains in the core of the bars can be estimated from the X-ray of the core of the bar, after shaving the skin, but as the peaks from PBT and PET overlap (see Figure S1b in Supplementary Information), deconvolution would be needed. However, the melting peaks of the two domains in Figure 6b do not overlap, hence the crystallinities of the PET and PBT domains were estimated from the heats of fusion using % Xc _PET_ = 100 × (ΔH_PET domain_/ΔH_100% PET crystal_) and % Xc _PBT_ = 100 × (ΔH_PBT domain_/ΔH_100% crystal PBT_). The DSC crystallinity value depends on the values of fusion for the 100% crystal, and there is a considerable range in the estimates for both polymers. We selected ΔH_100% PET crystal_ = 140 J/g (Wunderlich [25]) and ΔH_100% PBT crystal_ = 140 J/g (Illers [26]). Note that Illers gives a value of 166 J/g for ΔH_100% PET crystal_, but we selected the value of Wunderlich as it is closer to other reports. The per cent crystallinities of PET and PBT domains are given in Table 2.

### 3.4. Electrical Resistivity

Electrical conductivity is arranged in five ranges: insulator, anti-static (electrostatic dissipation, or ESD), conductive (EMI shielding), and conducting (metals, CNTs, etc.). The conductivity may be given in siemens/m or as volume resistivity in Ωm. The volume resistivity is the reciprocal of the conductivity. The surface resistivity is sometimes measured and is given in ohm sq. The volume resistivity is more a property of the material while the surface resistivity is influenced by other factors including contaminants.

**Figure 7:**
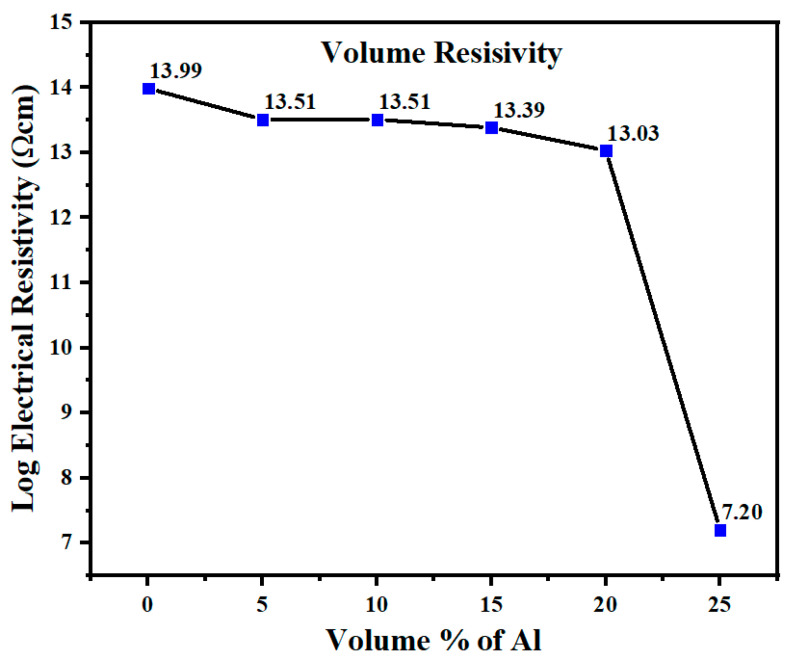
Electrical resistivity of 60/40 PBT/PET blend and Al nano platelet-filled 60/40 PBT/PET. The percolation threshold with Al nano platelets is 20–25 vol.%.

Figure 7 shows the volume resistivity of the unfilled 60/40 PBT/PET blend was 1 × 10^14^ Ωcm (or 1 × 10^12^ Ωm). For this blend with 5 to 20 vol.% Al content, there was a small decrease in volume resistivity from 1 × 10^14^ Ωcm for the unfilled blend; however, the values were still located in the insulating region at 1 × 10^13.5^ Ωcm to 1 × 10^13^ Ωcm (Figure 7). However, Figure 7 shows that between 21 and 25 vol.% of Al nano platelets, the electrical resistivity decreased greatly to a value of 7.2 × 10^7^ Ωcm (conductivity of 1.4 × 10^−8^ S/cm), from 1 × 10^14^ Ωcm for the 60/40 PBT/PET. The resistivity is now in the range of electrostatic dissipative plastics, but not in the EMI range. As mentioned in our previous work, when the same Al platelets as used in this work were placed in a single polymer, PET, even with 25 vol.%, the resistivity remained in the insulator range [18]. Figure 7 is in line with the expectation that a blend rather than a single polymer gives scope for the attainment of enhanced electrical conductivity.

Gao et al. [15] combined a hybrid filler of 10 phr of graphene + CNTs with a 70/30 PBT/PET blend. They cited an electrical conductivity of 9.61 × 10^−12^ S/m for their 70/30 PBT/PET (resistivity of 1.04 × 10^11^ Ω/m) but with 10 phr of CNTs, the electrical conductivity increased to 3.03 × 10^−3^ S/m (resistivity of 3.3 × 10^−4^ Ω/m); this put it into the EMI shielding range. However, 10 phr of graphene platelets alone in 70/30 PBT/PET led to a minor increase in conductivity (from 9.61 × 10^−12^ S/m to ~9 × 10^−12^ S/m). Thus, the rod-like CNT was more effective than the graphene platelets in the 70/30 PBT/PET blend.

Spitalskya et al. [27] gave a very extensive tabulation of the range of electrical conductivities achieved with CNTs in various polymer matrices. The values range from about 10^−5^ S/m (ESD range) to 3.89 × 10^2^ S/m (EMI shielding range), depending on filler content and processing methods. To get a perspective on where our Al-PBT-PET fits, Table 3 shows a selection of materials that span the five ranges of electrical conductivities [15,27,28,29].

### 3.5. Thermal Conductivity of Al-PBT-PET

**Figure 8:**
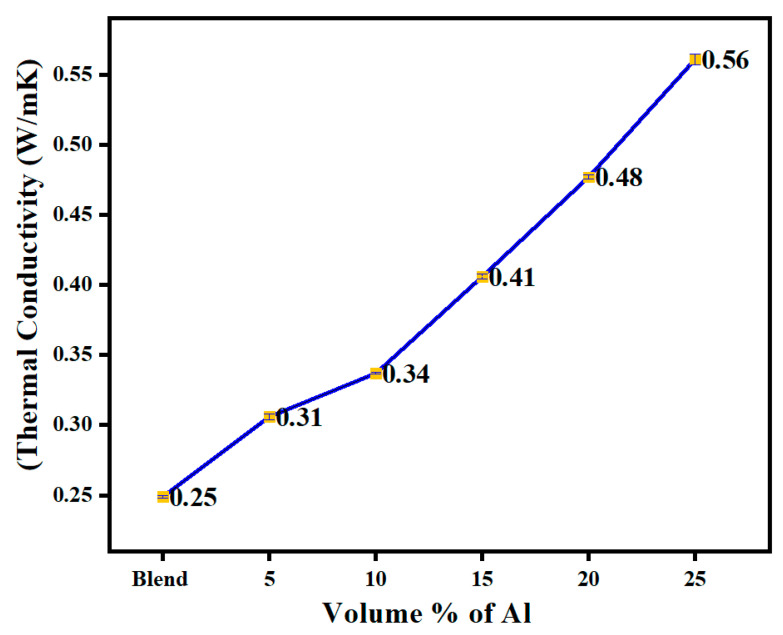
The thermal conductivity of Al nano platelet composites, at room temperature.

The thermal conductivity measurements of the PBT-PET blend filled with nano platelets of aluminium are shown in Figure 8. The volume fraction of 25 vol.% showed the highest thermal conductivity of 0.561 W/m·K which is a little over double the value of the 60/40 PBT/PET blend. Unlike electrical conductivity, where orders of magnitude changes can be observed at the percolation threshold, in thermal conductivity, the change even at percolation is smaller. Generally, filler loadings of 70 vol.% are needed to see percolation in thermal conductivity. Again, optimisation of the particle shape is needed for higher thermal conductivity at lower loadings [30]. Fibres or ribbons are better than flakes or spherical powders [31]. Danes et al. [32] showed that the thermal conductivity of PBT could be raised 10-fold from 0.22 W/m·K to 1.42 W/m·K, but it needed a 44 vol.% fraction of aluminium fibres (diameter = 90 μm, length = 1.1 mm). Table 4 shows the thermal conductivity of Al and plastics containing Al in various forms. Waheed et al. [33] reported an interesting work where acrylonitrile–butadiene–styrene filaments filled with synthetic micro diamond powder were 3D printed by the fused deposition modelling method. A thermal conductivity of only 0.94 W/m·K was attained with a loading of 60 wt.% of diamond powder, but the articles would almost certainly have been brittle (elongation-to-break of 1.7%). Nevertheless, interesting shapes for heat sinks were printed, and the work revealed the potential scope for applications for thermally conductive plastics [33].

Whether fillers with moderate thermal conductivity (alumina, ~27 W/m·K), high thermal conductivity (aluminium, 237 W/m·K), or super-high thermal conductivity (diamond, 2200 W/m·K; single CNT 3500 W/m·K; graphene 5000 W/m·K in the plane) are used, or hybrid filler strategies are employed, Guo et al.’s [35] review of thermally conductive plastics showed that a value between 1 and 5 W/m·K is mostly reached and it is difficult to exceed 10 W/m·K. Mechanical properties and price considerations are also limiting factors that decide the practicality. While 25 vol.% of Al nano platelets in PET was an electric insulator [18], and the same filler and amount when placed in a 60/40 PBT/PET blend transformed it into a conductive ESD plastic (Figure 7), Figure 8 and Table 4 show there was not as big an effect on thermal conductivity. The use of a blend with a co-continuous morphology can provide pathways for electrical conductivity (segregated network) at low volume fractions, but the same mechanism is not as effective for thermal conductivity.

### 3.6. Shrinkage Stability of the Injection-Moulded Articles of Al-60/40 PBT/PET above the T_g_

**Figure 9:**
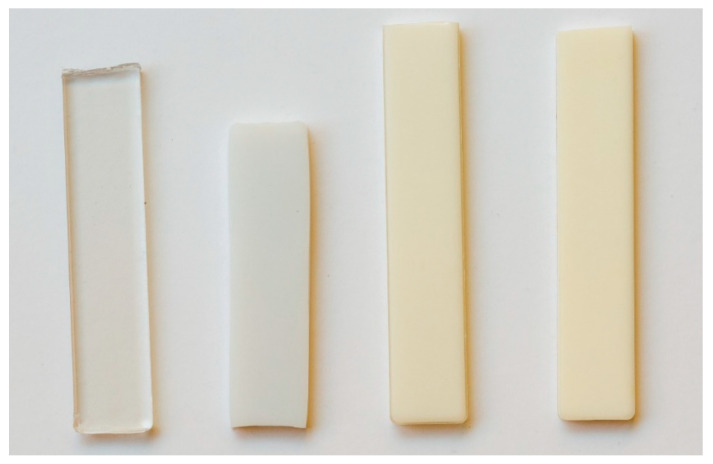
From the left, transparent amorphous PET bar; PET bar after annealing at 150 °C for 30 min. It cold crystallises, turns white, but shrinks and warps; as-moulded 60/40 PBT/PET bar is turbid and has a yellow tint, and shows a transparent amorphous skin; after annealing 60/40 PBT/PET bar at 150 °C for 30 min, the skin cold crystallises, but unlike the amorphous PET, there is no major shrinkage or warpage.

Table 5 shows the % shrinkage in length of as-moulded bars when annealed at 150 °C. The amorphous PET showed the highest shrinkage, as the density changed from 1.333 g/cm^3^ to 1.39 g/cm^3^ due to cold crystallisation. This magnitude of shrinkage is easily visible in the photo in Figure 9, and it led to gross and unacceptable distortion of the part. However, filling with 20 vol.% Al reduced the shrinkage substantially from 15% to 0.67% even with amorphous PET.

The 60/40 PBT/PET showed much less shrinkage than the as-moulded amorphous PET, which is also apparent in Figure 9, vindicating its selection as the matrix for a dimensionally stable conductive composite. In the 60/40 PBT/PET, the shrinkage of 0.67% was mostly due to the crystallisation of the transparent skin. The as-moulded bars of Al + 60/40 PBT/PET showed the lowest shrinkage of <1% at 150 °C (Table 5).

### 3.7. Mechanical Properties

Achievement of the conductivity target value is a necessary specification for a conductive plastic, but it is not sufficient for application and end-use. Often the problem is at the filler loading levels where percolation is reached, as there is an unacceptable drop in mechanical properties, especially the tensile strength and impact resistance. Many of the papers showing the rise in electrical and/or thermal conductivity after the addition of conductive fillers do not indicate the accompanying mechanical properties.

**Figure 10:**
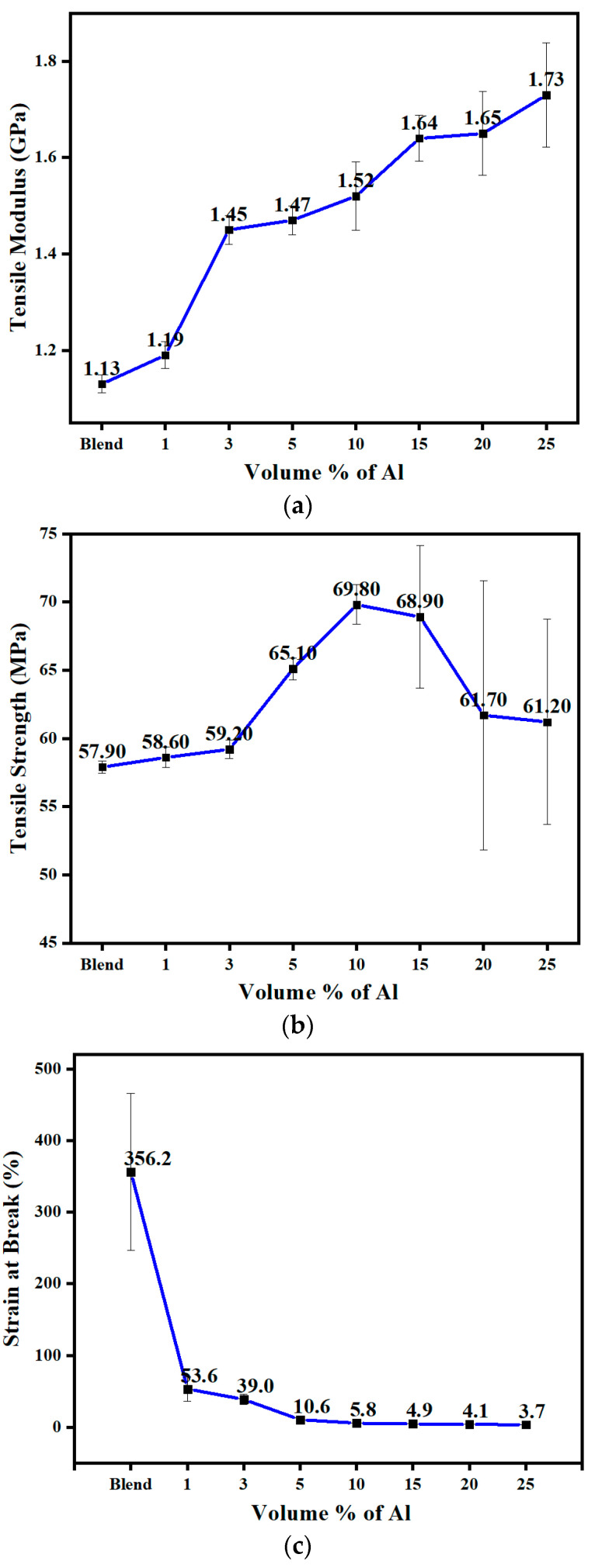
(**a**) The tensile modulus of 60/40 PBT/PET filled with aluminium nano platelets. (**b**) The tensile strength of 60/40 PBT/PET filled with aluminium nano platelets. (**c**) The strain at break of 60/40 PBT/PET filled with aluminium nano platelets.

Figure 10a shows the tensile modulus displaying an increase with the Al nano platelets, as is always the case when adding a rigid filler.

The tensile strength of unfilled amorphous PET, crystalline PBT, and the 60/40 PBT/PET was about the same (~60 MPa). In the 60/40 PBT/PET filled with Al platelets, Figure 10b shows that the tensile strength remained the same or displayed a small increase over PET and PBT, reaching a maximum of 69.8 MPa at 10 vol.% of Al, and then declining a little at higher loadings. At the highest loadings, the variation increased with some values falling below the matrix’s strength of 57.9 MPa, and this was due to increased agglomeration after about 20 vol.%. In most instances of filler–polymer systems, the tensile strength decreases monotonically with increasing filler content by about 1/3 to 1/2 compared with the unfilled polymer [36,37]. This is due to poor adhesion of the matrix and the filler.

Figure 10c shows the 60/40 PBT/PET had a high elongation-to-break of 356%. This is much higher than many plastics where the elongation-to-break is less than about 50%. However, the addition of any rigid filler always exponentially reduces the elongation-to-break as the filler immobilises the chains [38]. This is also seen with the 60/40 PBT/PET filled with the Al platelets in Figure 10c. Just adding 1 vol.% of the Al flakes caused a drop in the elongation-to-break to 54%.

**Figure 11:**
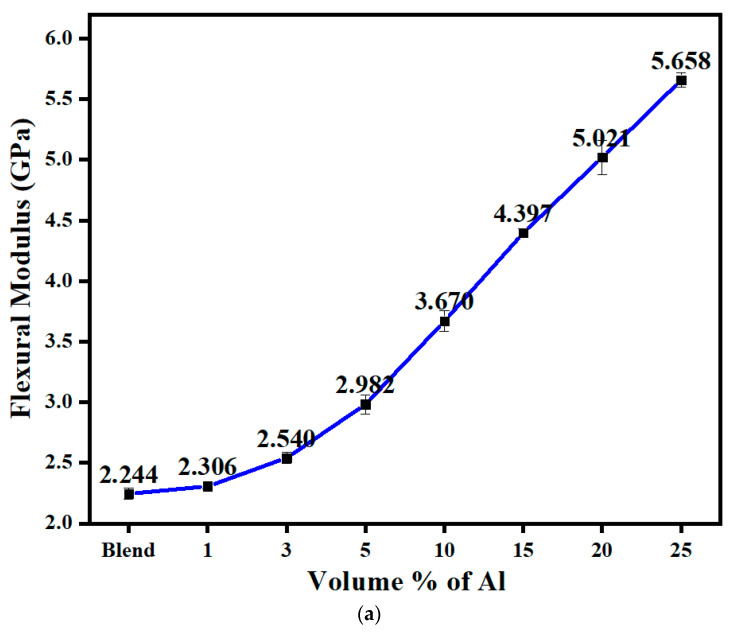

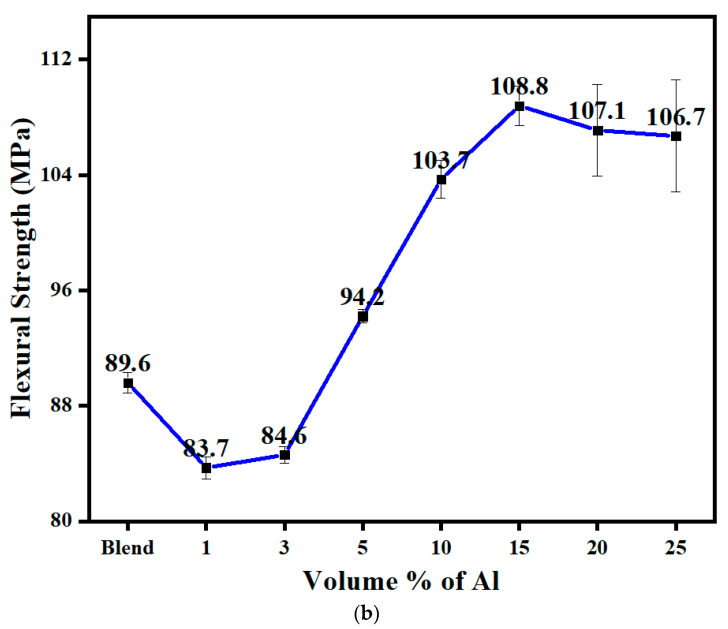
(**a**) Flexural modulus of 60/40 PBT/PET filled with aluminium nano platelets. (**b**) The flexural strength of 60/40 PBT/PET filled with aluminium nano platelets.

Figure 11a shows the flexural modulus of the 60/40 PBT/PET as a function of the volume % of nano Al platelets. The flexural modulus showed a marked rise with platelet content and this was due to the tendency of the platelets to become oriented parallel to the surface of the bar. The highest value obtained was 5.66 GPa with 25 vol.% of Al platelets; with the same in PET, a flexural modulus of 8 GPa was attained [18].

Figure 11b shows the flexural strength was in the range 84–92 MPa for all the bars. The flexural strength declined slightly below the matrix’s value at the lowest loadings of Al (3 vol.% and lower), but rose at higher loading above the blend’s value. The dip was real as it was based on 10 measurements. The most probable cause is that at 3 vol.% and lower, the platelets had less preferential orientation. At Al contents >3 vol.%, the flexural strength increased as the platelets took up orientation parallel to the main (widest) surface of the bar (shown by microscopy). At 20 and 25 vol.%, higher variability was detected as with the tensile strength, however, even with this, the flexural strength was clearly well above that of the matrix. The orientation of the platelets parallel to the bar’s surface had a greater effect on the strength in flexure compared with tension, and the effect of agglomeration at the highest loadings had less effect on the variation in flexural strength than in tensile strength.

The impact resistance is often a determining factor in engineering use, and hence we measured the values for amorphous PET, crystalline PBT, and crystalline 60/40 PBT/PET, and then the 60/40 PBT/PET with Al flakes. For amorphous PET, we measured a notched Izod impact resistance of 24 J/m. Tanrattanakul et al. noted that crack initiation in PET is actually difficult, but once notched, crack propagation is fast [39]. For PBT, we measured a value of 53 J/m. Aravinthan and Kale [40] reported a value of 25 J/m for PET and 27 J/m for PBT; that is, in their work both polyesters had similar impact resistance. We do not know why we obtained a higher impact value than Aravinthan and Kale for PBT. Our 60/40 PBT/PET blend showed a value of 34 J/m. That is, it is midway between the constituents, PET and PBT. In contrast, Aravinthan and Kale [40] reported a value of 42 J/m of the 60/40 PBT/PET blend, that is, an increase over both their constituents, whereas here, in the blend, there is an increase in the impact resistance relative to PET but a decrease with respect to PBT.

**Figure 12:**
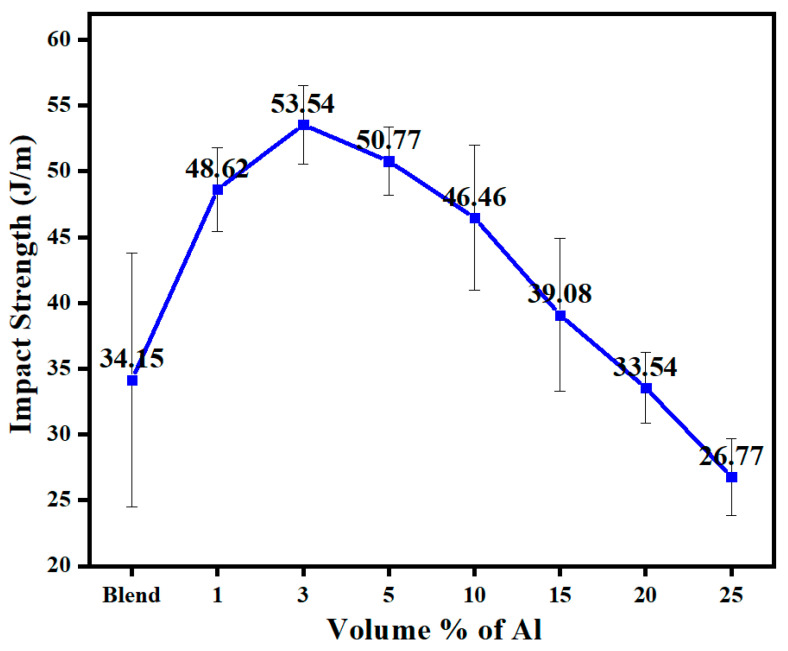
The impact resistance of 60/40 PBT/PET filled with aluminium nano platelets.

Figure 12 shows the effect on the notched Izod impact resistance when the 60/40 PBT/PET blend was filled with nano Al platelets. There was a rise in impact resistance relative to the unfilled blend followed by a decrease. The maximum of 53.5 J/m occurred at 3 vol.% and is comparable to the value of the PBT here. At the higher loadings, such as 25 vol.% of Al, the impact resistance was decreased below the value of the 60/40 PBT/PET, but it still remained comparable (26.8 J/m) to one constituent polyester, the PET (24 J/m).

In summary, the Al-PBT-PET blends have mechanical properties that are in a usable range. However, Figure 12 shows it is important to achieve the electrical percolation at lower volume percent to get the optimum impact resistance, and this requires the optimisation of the filler shape.

### 3.8. Co-Continuous Morphology in the 60/40 PBT/PET Blend

**Figure 13:**
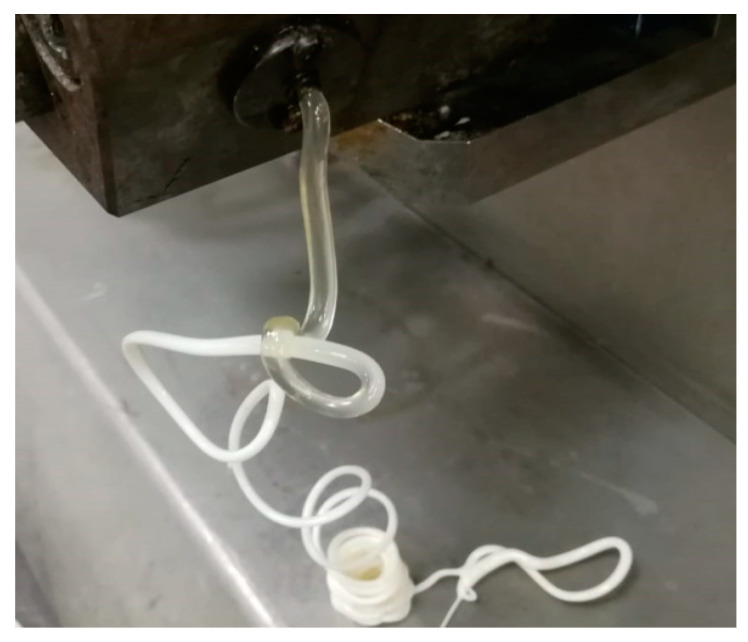
A 60/40 PBT/PET melt emerging from the die is transparent, indicating that the components are either miscible in the melt, or the constituents are in nano domains with similar refractive indices. On cooling, whitening occurs due to crystallisation.

While it is known that PBT-PET blends are not miscible in the solid state because they crystallise separately, as confirmed by the two melting peaks corresponding to PBT and PET domains (Figure 6), we observed that the PBT-PET melt exiting from the die was transparent (see Figure 13), and turbidity only sets in during crystallisation. This was intriguing and unlike most other blends, such as polypropylene (PP)-PET, where the melt exiting the die is itself turbid, due to light scattering from micron-sized domains of one polymer in the other. The transparency of the PBT-PET blend in Figure 13 at the die face could mean the components are miscible in the melt, in which case, they have to de-mix very rapidly as it cools, before crystallisation can take place in two domains; or, it could be that the melt still has domains of the two polymers, but they are nano in size and have refractive indices that are matched, so it appears transparent. We shall attempt to shed light on this from the SEM of the fracture surface of the 60/40 PBT/PET bars.

**Figure 14:**
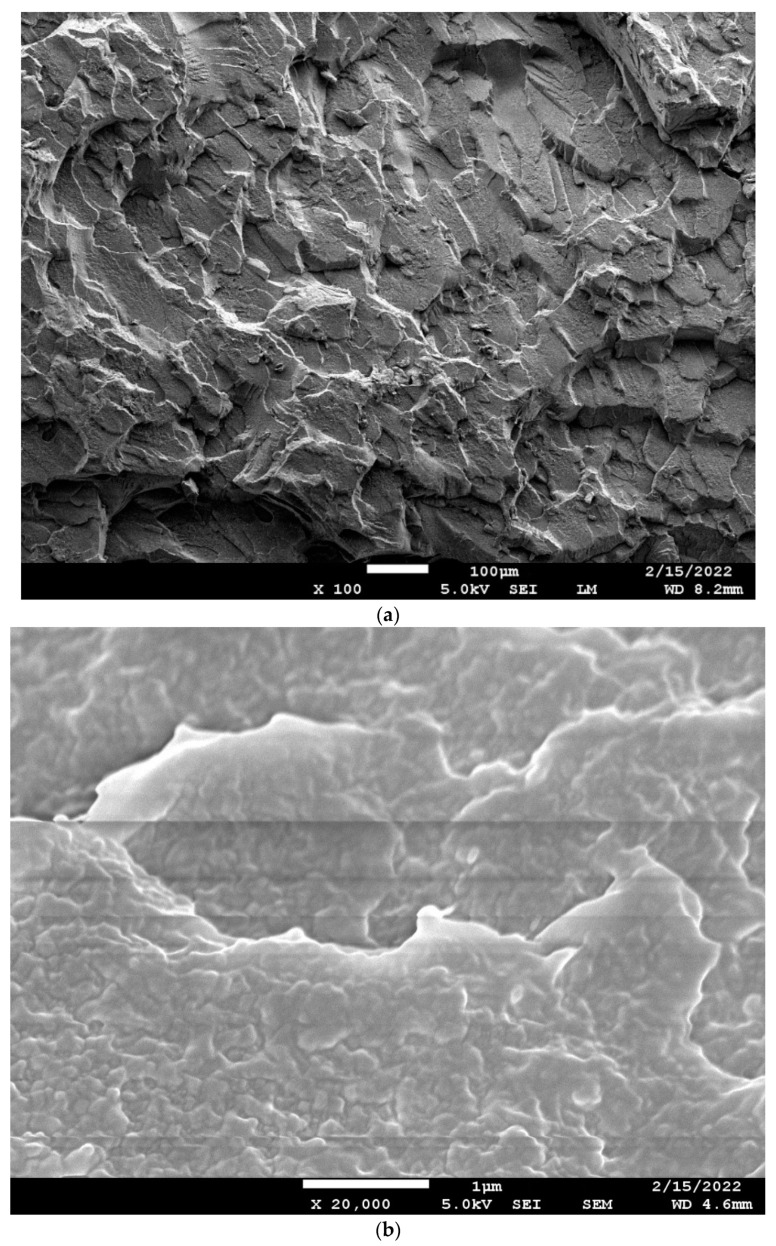
(**a**) Low magnification picture of cryo-fracture surface of 60/40 PBT/PET bar. The white size bar = 100 μm. (**b**) High magnification of the cryo-fracture surface of 60/40 PBT/PET bar shows the interpenetrating co-continuous domains of PET and PBT. The white size bar = 1 μm. Unlike most other polymer blends, the domain dimensions are sub-micron.

Figure 14a shows the SEM picture of the cryo-fracture surface of the 60/40 PBT/PET tensile bar. This does not show any indication of the expected co-continuous morphology. A high magnification (Figure 14b) was needed to reveal there are domains, at least in the solid state, and these have the classical co-continuous morphology where the two domains are not discretely separated likes spheres embedded in a matrix. Willemse et al. [41] give a definition of ‘co-continuous morphology’ whereby both phases form a single, continuous, interpenetrating structure, and they explained the geometrical conditions for the minor phase to become continuous. The interfacial tension and the melt viscosity ratios determine the volume fractions where co-continuous morphology can form [41]. Figure 14b shows that the PET and PBT domains form a classical co-continuous morphology but the phase domains were <1 μm (about 500 nm or lower in size); the contrast was low and it is difficult to say which is PET and which PBT. In other works investigating the co-continuous morphology, the blend was treated with solvent to etch out one component, to increase the contrast [41]. However, PET and PBT have few solvents, and the ones that can dissolve would attack both, hence we did not attempt to improve the contrast in Figure 14b by selective etching. The PBT and PET can trans-esterify, which if taken to the limit can lead to random co-polymers. However, the trans-esterification is slow and it does not take place in a major way in the time scale of the melt compounding and injection moulding; otherwise, if a random co-polymer had been made, one would not see the melting peaks of PBT and PET as in Figure 6. Infrared spectroscopy (not shown) of the blend did not show any conversion to co-polymer. However, it is likely that a low level of trans-esterification does take place, and this self-compatibilisation would further blur the boundaries between PBT and PET nano domains, and reduce the contrast, as in Figure 14b.

In our experience of other blends such as PP-PET, in compositions such as 60:40 and 50:50, a co-continuous morphology with sharper contrast is obtained even without selective etching, and the domain sizes are in the range of 5–10 μm instead of 500 nm. Li et al.’s work [10] on carbon black in PA6/PBT blends showed a very good example of a co-continuous morphology with 50:50 PA6:PBT; contrast was improved by etching the PBT component with alcoholic KOH. However, the phase domains were tens of microns in width, rather than 500 nm or lower as in 60/40 PBT/PET (Figure 14b). Likewise, Chang et al. showed the sea-island and co-continuous morphology of PBT-polylactic acid blends [41,42]. Again, the domain width was about 10 μm and not 500 nm as in PBT-PET. Likewise, micrographs of 35 vol.% polystyrene in polyethylene, and 37 vol.% polyethylene in PP [41], show co-continuous morphologies where the phase dimensions are in microns. In most other immiscible blends, the co-continuous morphology can be seen at a magnification of about 2000× but, for PBT/PET blends, it will be missed unless a magnification of 20,000× is used as in Figure 14b. Willemse et al. [41] noted that the effect of increasing the interfacial tension will be an increase in the phase dimensions; conversely, the small domain sizes of the PBT and PET imply low interfacial tension.

Aravinthan and Kale [40] investigated the fracture sections of the complete range of compositions of PBT/PET blends. Although they stated that the 40/60, 50/50, and 60/40 PBT/PET compositions had co-continuous morphologies, their SEM pictures did not demonstrate the typical appearance. They used a lower magnification and, according to us, the co-continuous structure cannot be seen as the phase domains are small in size (Figure 14b). Gao et al. [15], who used a 70/30 PBT/PET blend with hybrid graphene + CNT fillers, stated that there was no observable phase separation of the polymers and they concluded there was ‘good compatibility’ between the PET and PBT.

There are ambiguous conclusions on the miscibility of PBT/PET blends; partial miscibility and even total miscibility in the melt has been suggested, with possible dependence on molecular weight. Arivanthan and Kale [40] thought that the PET and PBT are miscible over the entire composition range. Shonaike [43] conducted DSC studies on PBT-PET blends and concluded the blends were miscible in the amorphous state. We also recall Avramova’s [24] work with ultra-quenched PBT-PET blends. Based on DSC studies, where a single T_g_ was seen, she concluded that the PBT-PET blends were miscible in the amorphous state at all compositions (and implicitly in the melt state). She stated that both polymers crystallised simultaneously at all compositions and each component formed its own crystal phase. This implies rapid de-mixing has to take place before cold crystallisation, which would be even more remarkable as it means de-mixing would take place from the amorphous glass which has a higher viscosity than the melt. The co-continuous morphology of Figure 14b suggests that there should be two T_g_s, but this is not seen. The T_g_ of the PBT is seen at ~50 °C in Figure 6a, but the T_g_ of the PET domains at ~78 °C is obscured by the broad cold crystallisation exotherm of the PBT which occurs over it (arrow in Figure 6a). The way to resolve this would be through modulated DSC.

From the co-continuous morphology of Figure 14b, we hypothesise that it is more likely that the transparent 60/40 PBT/PET melt in Figure 13 is not a homogeneous, single phase; despite the transparency, it is likely to have nano domains of PBT and PET, but there could be refractive index matching. In this case, crystallisation of PET and PBT can take place without rapid de-mixing.

### 3.9. Fracture Surfaces of 60:40 PBT:PET Blend with Al Platelets

**Figure 15:**
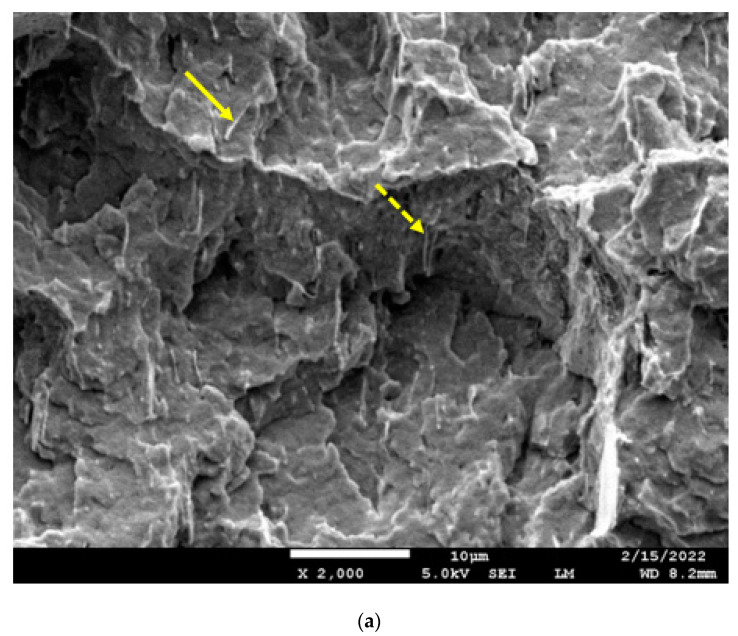

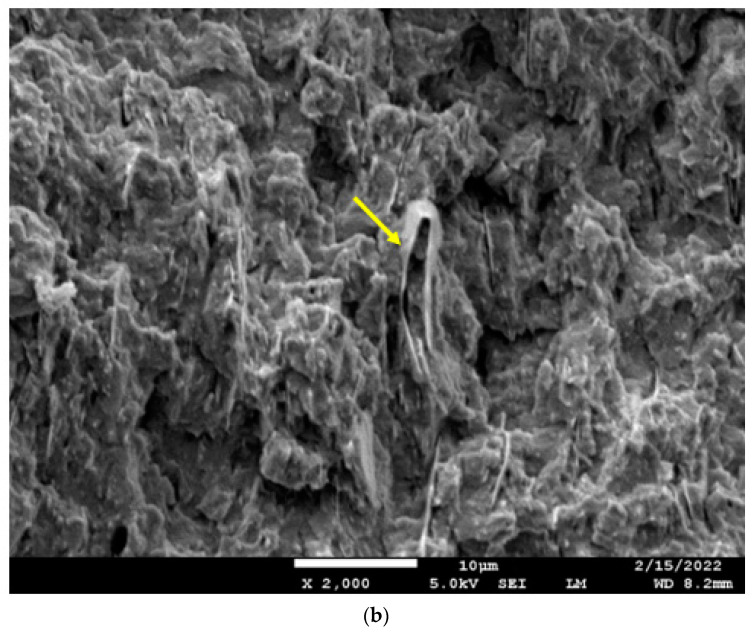
(**a**) SEM image of the cryo-fracture surface of a bar of the 60/40 PBT/PET blend with 10 vol.% Al flakes. The platelets are seen edgewise. The rectangle in the left corner is the cross-section of the bar (not to scale) and is placed to visualise the platelet orientation relative to the bar. The arrow shows polymer coating on a platelet. Dotted arrow shows a folded platelet. The white size bar = 10 μm. (**b**) SEM image of the cryo-fracture surface of a bar of the 60/40 PBT/PET blend with 25 vol.% of Al nano platelets. The platelets are seen edgewise. The arrow shows a folded platelet with polymer coating. The white size bar = 10 μm.

Figure 15a shows the cryo-fracture section of 60/40 PBT/PET containing 10 vol.% of Al platelets. The platelets are only seen edge-on in the fractured section of the bar, indicating they become oriented parallel to the flow. To visualise the orientation of the platelets relative to the bar, it is indicated that we are imaging a central region of the bar’s rectangular cross section in Figure 4, but with the section rotated by 90°. It can also be seen that the platelets lie parallel with each other and all in one direction. Additionally, some of the larger Al platelets show folding. In all compositions, the Al platelets were not pulled out of the blend matrix. Where an Al platelet protrudes (arrow in Figure 15a), it is coated with polymer. This can be contrasted with Al platelets in a PP matrix [37] or graphene in PBT/PET [15], where platelets are pulled out and show no polymer coating. The bold arrow in Figure 15a indicates polymer covering a platelet, indicating the adhesion between the particles and the blend is good. That was the feature observed with Al and PET [17,18], and it also extends to 60/40 PBT/PET blends. The dotted arrow in Figure 15a shows a platelet with a large length that folded. This can be contrasted with Gao et al.’s graphene nano platelets in a 70/30 PBT/PET blend; besides pull-out of graphene platelets without polymer coating, they noted there was size reduction due to breakage by shear action of the screw [15]. The Al nano platelets are more ductile and fold.

It was stated that we would use the concept of a ‘segregated network’ in a polymer blend to achieve conductivity. While a drop in electrical resistance was observed between 20 and 25 vol.% of Al platelets in the 60/40 PBT/PET blend in Figure 7, which was not seen when the same Al platelets were placed in PET, and this would be consistent with the proposal of Sumita et al. [9] and the observations of others that higher conductivity is possible with an immiscible blend than in a single polymer, the SEM picture in Figure 15b shows that the segregated network is difficult to see in our case when the large platelets are present. Mamunya et al. [7] had shown that on compression moulding of poly(vinyl chloride) powder with metal particles, the condition for the formation of the ‘segregated network’ was that the conductive particle should be very much smaller in diameter than the polymer particle. Likewise, in cases where a segregated network was achieved with conductive particles in a polymer blend, such as the work of Li et al. [10] using carbon black in a PA6/PBT blend, the conductive particles (carbon black) had dimensions of ~100 nm while the polymer domains in the blend (whether it was sea-island or co-continuous) were typically several microns in size. This allowed the conductive particles to nestle in the regions between domains. In our case, we have a case where the co-continuous morphology has unusually finer domains (sub-micron) compared with other blends, while the platelets are much larger (up to 10 μm in lateral width). There appeared to be a higher number of folded and bent platelets compared with Al-PET [18] and this could be because the Al platelets were too big to fit between the PBT-PET nano domains. Higher magnifications could not clearly show the platelets nestling between sub-micron domain boundaries. It would suggest that Al nano spherical particles placed in a 60/40 PBT/PET blend with a co-continuous morphology might be better for achieving a ‘segregated network’ than Al platelets.

## 4. Conclusions

In our previous works, we had used nodular Al and Al platelets in a single polymer (amorphous PET) matrix. The adhesion between Al and PET was good, hence the mechanical properties were good; however, even at 25 vol.% of Al, the material remained an electrical insulator. In this work, we explored the possibility of attaining a conductive composite with Al platelets by placing them in a 60/40 PBT/PET blend. Such a blend should lead to a co-continuous morphology which the literature had indicated would be conducive to the double percolation mechanism whereby the conductive particles cluster at domain boundaries and form a segregated electrically conductive network. Further, the use of a 60/40 PBT/PET blend instead of PET would also allow the attainment of a crystalline matrix, so that moulded articles would show stability against shrinkage above the higher of the two T_g_s. Finally, it was hoped the adhesion would not be grossly impaired leading to drastic reduction in mechanical properties.

With Al nano platelets in a 60/40 PBT/PET blend as the matrix, the results show that a reduction in electrical resistivity by ~6 orders (7.2 × 10^7^ Ωcm from 1 × 10^14^ Ωcm, corresponding to a plastic capable of electrostatic charge dissipation) was reached with 20–25 vol.% of Al platelets. This would be in line with other cases where a decrease in electrical resistivity is observed in co-continuous immiscible polymer blends rather than in single polymers, using the same filler. However, the co-continuous morphology in 60/40 PBT/PET blend was not readily apparent, because unlike other polymer blends, the domain sizes were finer and the contrast was poorer. Previous works using PBT/PET blends had not demonstrated the co-continuous morphology as they used lower magnifications, under which the co-continuous feature in PBT/PET blends would not be detectable. Earlier works on PBT/PET blends suggest partial or even total miscibility. However, miscibility in our view is problematic as the separate crystallisation of the two components would require a rapid de-mixing before this can happen. The transparency of the PBT/PET blend is suggestive of miscibility, but this work hypothesises it could be because the PET and PBT domains are of nano size and if the refractive index is similar, then transparency is possible. Due to the unexpectedly finer scale of the co-continuous morphology in the 60/40 PBT/PET blend, the Al flakes used here might not be the best for aggregating between the domain boundaries, as the platelets (though nano in thickness) were 5–10 μm wide.

The thermal conductivity increased by 2x at 25 vol.% of Al platelets. Unlike electrical conductivity, thermal conductivity requires much higher loadings, and using a co-continous blend in this case does not seem to have any benefit. The mechanical properties were in a range that is usable: the tensile modulus increased, and the tensile strength showed an increase. The impact resistance decreased compared to unfilled blend, but was about the same as amorphous PET (24 J/m). That is, an electrically conductive composite with ESD capability, usable strength and impact resistance, and crystallisation during injection moulding time scales can be made with an injection-mouldable polyester composition of Al-60/40 PBT/PET as the matrix. The articles moulded from this composition showed a low shrinkage of under 1% when heated to 150 °C, compared with Al–amorphous PET which was over 1%. Thus, the 60/40 PBT/PET is a better and a more convenient matrix for electrically conductive plastics than Al–amorphous PET. Finally, for a conductive plastic, the price of the filler should not be exorbitant, and there should be ease of operation in manufacture without complicated modifications of the polymer or the filler’s surface. This is one of the current drawbacks of using CNTs and graphene. Al with polyester matrices avoids this complication.

The Al-PET-PBT composition can now be further optimised, and one can aspire to reduction in resistivity to EMI levels. One optimisation that needs to be done is to reduce the volume fraction of Al at which electrical percolation starts from 20–25%. This would simultaneously increase the impact resistance. This work suggests that spherical Al nano particles would be better than Al platelets for the creation of the ‘segregated network’ in 60/40 PBT/PET blends. This reduction in the volume fraction needed for conductivity should also be possible by using chopped Al micro fibres. Further, increase in impact resistance can be achieved by adding rubber tougheners. In some applications, flame retardants would also be needed. Cost reduction is possible by using recycled PET which is readily available.

## Figures and Tables

**Table 1:** Formulations of the Al nano platelet composites in a 60/40 PBT/PET matrix. The PBT/PET blend’s density was 1.3192 g/cm^3^ while Al has a density of 2.70 g/cm^3^.

Sample ID	Composition of Al Platelets in the Blend (vol.%)	Composition of Al (wt.%)
PBT-PET blend	0% Platelet al. (0) + PBT/PET (60/40) (100)	0.00
01 vol.% Platelet	01% Platelet al. (01) + PET/PBT (99)	2.03
03 vol.% Platelet	03% Platelet al. (03) + PET/PBT (97)	5.95
05 vol.% Platelet	05% Platelet al. (05) + PET/PBT (95)	9.72
10 vol.% Platelet	10% Platelet al. (10) + PET/PBT (90)	18.53
15 vol.% Platelet	15% Platelet al. (15) + PET/PBT (85)	26.53
20 vol.% Platelet	20% Platelet al. (20) + PET/PBT (80)	33.85
25 vol.% Platelet	25% Platelet al. (25) + PET/PBT (75)	40.56

**Table 2:** Xc is the % crystallinity estimated from DSC of the 60/40 PBT/PET blend and aluminium-filled versions.

Material	% Xc PET Domains	% Xc PBT Domains
PBT pellet	-	24.70
PET pellet	22.4	-
60/40 PBT/PET	28.71	19.63
1% Al + 60/40 PBT/PET	24.50	16.43
3% Al + 60/40 PBT/PET	23.96	16.78
5% Al + 60/40 PBT/PET	28.72	21.64
10% Al + 60/40 PBT/PET	26.37	22.50
15% Al + 60/40 PBT/PET	24.48	22.98
20% Al + 60/40 PBT/PET	17.36	20.53
25% Al + 60/40 PBT/PET	11.63	21.47

**Table 3:** Resistivity and conductivity at 20 °C of a range of materials.

Material	Resistivity (Ωm) at 20 °C	Conductivity (S/m) at 20 °C	Type	Reference
Silver	1.59 × 10^−8^	6.30 × 10^7^	Conductor	[28]
Aluminium	2.82 × 10^−8^	3.5 × 10^7^	Conductor	[28]
Carbon (graphene monolayer film)	2.89 × 10^−7^	3.46 × 10^6^	Conductor	[29]
Carbon multi-wall nanotube film	1.425 × 10^−6^	7.065 × 10^5^	Conductor	[29]
Silicon	2.3 × 10^3^	4.35 × 10^−4^	Semi-conductor, depends on added impurities	[28]
CNT composites	10^5^ to 2.6 × 10^−3^	10^−5^ to 3.89 × 10^2^	Conductive (ESD to EMI shielding)	[27]
10 phr of CNTs in 70/30/PBT/PET blend	3.3 × 10^−4^	3.03 × 10^−3^	Conductive (EMI shielding)	[15]
Al-PBT/PET	7.2 × 10^5^	1.4 × 10^−6^	Conductive (ESD)	This work
PBT/PET (plastics)	10^12^	10^−12^	Insulator	This work

**Table 4:** Thermal conductivity of plastics filled with different types of aluminium.

Material	Thermal Conductivity (W/m·K)	Matrix and Filler	Reference
Al	237	-	Carvill [34]
9 vol.% carbon black in PBT	0.37	Single polymer, single filler	Danes et al. [32]
25 vol.% Al powder in PBT	0.79	Single polymer, single filler	Danes et al. [32]
44% Al micro fibres in PBT	1.42	Single polymer, single filler	Danes et al. [32]
9% Al powder + 9% Al fibre + 2% carbon black in PBT	0.5	Hybrid filler, single polymer	Danes et al. [32]
25% Al platelets in amorphous PET	0.606	Single polymer, single filler	Arfat et al. [18]
25% Al platelets in crystalline 60/40 PBT/PET	0.561	Single filler, co-continuous blend	This work
Commercial conducting plastics	~1–10		

**Table 5:** Change in the length of the moulded bars (cut flexural bar) after annealing at 150 °C.

Moulded Bar	Length Initial (mm)	Length Final (mm)	Standard Deviation	Change (mm)	% Shrinkage
Amorphous PET	60	51	1.27	9	15.00
20% Al platelets amorphous PET	60	59.60	0.14	0.40	0.67
60/40 PBT/PET	60	59.60	0.28	0.40	0.67
20% Al platelets + 60/40 PBT/PET	60	59.90	0.07	0.10	0.17

## Data Availability

Data is contained within the article or Supplementary Materials.

## Supplementary Materials

The following supporting information can be downloaded at: https://www.mdpi.com/article/10.3390/polym14061092/s1.
