# Supplementary material for: Conductive Plastics from Al Platelets in a PBT-PET Polyester Blend Having Co-Continuous Morphology
Source: Polymers (Basel). 2022 Mar 9;14(6):1092. doi: 10.3390/polym14061092 (PMC8951038; doi:10.3390/polym14061092)
Supplement: Supplementary file 1 [file polymers-14-01092-s001.zip › polymers-1545972-supplementary.pdf]

Supplementary Information

Tensile testing according to ASTM D638-14 (Standard Test Method for Tensile Properties of Plastics)

The tensile bars had the following dimensions: end-to-end length L = 150 mm, width W = 12.7 mm × Depth D (thickness) = 3.25 mm. The length of the narrow section of the bar was 57 mm and the gauge length was 55 mm. The radius of curvature of the curved portion (fillet) which joins the narrow part of the bar to the grip portion was 76 mm. Our tensile bar's dimensions correspond in all details to the Type I bar of ASTM D638-14 (Standard Test Method for Tensile Properties of Plastics), except in the ASTM Type I, the end-to-end length L = 165 mm. That is, in our tensile bar, the grip section was shorter than in the ASTM bar by 7.5 mm, but this has no influence on the measurement, and as all other features including the gauge length are the same, we say our tensile bar was in essence the same as the Type I tensile bar of ASTM D638-14. The reason the grips were made shorter was because of the limitation of the shot size for the mini-injection moulder.

The cross-head speed used was 50 mm/min. ASTM D638-14 allows 5, 50 or 500 mm/min., and recommends the slowest according to the time to reach the extension to break (< 5 mins.). For the 60/40 PBT/PET bar if 5 mm/min. is chosen, the extension-to-break would be > 356 % and the time of the test would be too long. The intermediate value of 50 mm/min. was thus selected. This kept the extension-to-break under 5 minutes for the samples. We are interested in the relative modulus between the blend and the Al-filled blend, hence a common cross head speed of 50 mm/min. was used for all.

No humidity conditioning was used. The bars were prepared through injection moulding, stored in plastic bags and tested several days after moulding. They were kept in an air-conditioned laboratory at 20°C. The 60/40 PBT/PET was not as sensitive as polyamides and cellulose to moisture uptake. Again, this factor did not affect the relative modulus and the trend obtained was as expected. The number of samples was 10; all breaks were in the gauge length.

The tensile bar was injected from one end of the bar. For the Al platelets, there was anisotropy at loadings > 3 vol.% of Al - in this case the platelets would align with the flow direction; that is be edge-on if the tensile bar's section was viewed.

Flexural test according to ASTM D790-03 ('Standard Test Methods for Flexural Properties of Unreinforced and Reinforced Plastics and Electrical Insulating Materials), Procedure A

This was used to measure the flexural modulus and flexural strength of the unfilled and Al filled 60/40 PBT/PET.

Rectangular bars were injection moulded according to the dimensions of ASTM D790-03. As with the tensile tests, no humidity conditioning was used. The bar's length was 134 mm, the width was 12.7, and the depth (thickness) was 3.25 mm. The bars were tested flatwise rather than edgewise. The ASTM standard says that a support span-to-depth ratio of 16:1 shall be used. The span is the distance between the supports on which the bar rests. As the depth was 3.2 mm, the span was measured and set at 51.2 mm. As the bar's length was 134 mm, this left an overhang of 41.4 mm on either side of the support (ASTM D790-03 specifies an overhang of at least 10% of the support span which would be 5.12 mm, and no less than 6.4 mm). The span distance was kept constant for the bars. The bar was places on the supports such that the loading nose contacted in the middle of the bar. The radii of the loading nose and supports was 5.0 mm as specified in the standard. The cross-head speed was 2 mm/min, the calculated value of rate of crosshead motion was 1.365 mm/min. The ASTM D790-03 recommends a test speed range calculated depending on the support span, the depth of the beam and strain rate on the outer layer. The crosshead speed R should be set by $R = ZL^2/6d$; where L = support span in mm, d = depth of beam in mm, and Z is the rate of straining of the outer layer, in mm/mm/min. The standard specifies Z to be 0.01 and says that in no case shall the actual crosshead speed differ from that calculated from the equation, by more than 610 %. The calculated speed according to our choice of span (L = 51.2 mm) and depth (3.2 mm) was 1.365 mm/min., and our chosen speed of 2 mm/min. falls within the standard's range of 610% difference.

In composites, the orientation of the reinforcement can influence the flexural modulus and strength. With the Al nano platelets, microscopy of fractured tensile bars established they were seen only edge-on at loadings > 3 vol. %, implying the platelets were oriented with their flat surface parallel to the widest surface of the bar.

The number of tests was 10 per composition. The tangent flexural modulus was calculated by drawing a tangent to the steepest initial straight-line portion of the load-deflection curve and using the following equation, $E_B = L^3 m/(4bd^3)$, where, E_B = modulus of elasticity in bending, MPa, L =

support span, mm, b = width of bar tested, mm, d = depth of bar, mm, and m = slope of the tangent to the initial straight-line portion of the load-deflection curve, N/mm of deflection. No toe correction was applied.

Notched Izod Impact Resistance according to the ASTM D256-04

The impact resistance of the Al filled-60/40 PBT/PET bars was compared with the 60/40 PBT/PET bar, by using the notched Izod test according to the ASTM D256-04. The moulded bar's dimensions were length of 64 mm, width of 12.7 mm and thickness of 3.25 mm as set by the standard. It was notched by machine. The V shaped notch was cut at the mid-point along the length of the bar (31.8 mm) and had full cone angle of 45 degrees and was 2.5 mm deep (radius). The distance in front of the notch in the bar was = 12.7-2.5 = 10.2 mm.

The standard has four methods of tests (A, C, D and E), and three types of break and one non-break as outcome. The standard says Test Method C is preferred over Test Method A for materials that have an Izod impact resistance of less than 27 J/m under the notch. In test method C a correction is applied to account for the toss energy (to propel the broken fragment) which is a substantial fraction of the energy for low impact samples. 25 J/m is on the border between Type A and C; however, the bars had values > 27 J/m, hence we decided to use Type A (no toss correction) for both the 60/40 PBT/PET and the Al-60/40 PBT/PET composites. The test was conducted notch wise (pendulum striking on the notch side).

The blend and all the Al-blend bars broke according to the type C (Complete Break) break mentioned in the standard - that is on break, the bar separated into two or more pieces. Hence, impact value comparisons are valid as the failure category was the same for all compositions.

The bars were stored for over 40 h at 23°C but not conditioned for humidity. At 23°C, the /40 PBT/PET is not as hygroscopic as polyamides and cellulose.

X-ray diffractograms (XRD) of 60/40 PBT/PET bars (as moulded, after shaving skin, and after annealing)

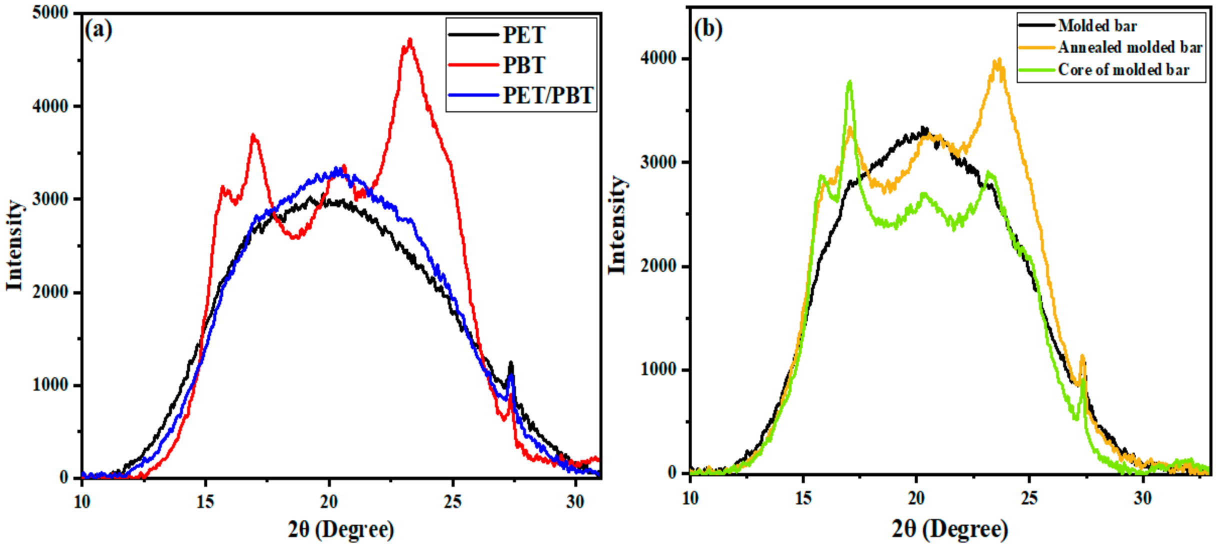

Figure S1: XRD of (**a**) superimposed curves of as-molded PET, PBT and 60/40 PBT/PET bars (**b**) superimposed curves of as-molded 60/40 PBT/PET bar (black), the as-moulded bar after annealing at 170°C for 30 mins. (yellow) and the core of the 60/40 PBT/PET bar after shaving off the transparent skin. Annealing causes crystallization of the amorphous skin, so overlapping peaks of crystalline PBT and PET are seen. Shaving reveals, the core of the as-moulded 60/40 PBT/PET bar has the same overlapping peaks of crystalline PBT and PET, indicating the core crystallizes during molding. The small sharp peak at 2θ ~ 27° is an artefact.

The Figures above confirm that (1) there is a skin in the as-molded 60/40 PBT/PET bar as also apparent by visual inspection (2) the skin is amorphous 60/40 PBT/PET and (3) the core of the bar has crystallized PBT and PET.

The main peaks of annealed (170°C and 30 mins.) 60/40 PBT/PET bar are at 2θ 16.11°, 17.30°, 21.15°, 23.41° and 25.18° while the main peaks of annealed PET (170°C and 30 mins) are at 16.58°, 17.64°, 21.74°,22.84° and 26.02°. The main peaks of molded PBT are at 2θ 15.70°, 16.91°, 20.62°, 23.27°.
